# Endocytosis of DNA-Hsp65 Alters the pH of the Late Endosome/Lysosome and Interferes with Antigen Presentation

**DOI:** 10.1371/journal.pone.0000923

**Published:** 2007-09-26

**Authors:** Ana Paula F. Trombone, Célio L. Silva, Karla M. Lima, Constance Oliver, Maria Célia Jamur, Alan R. Prescott, Arlete A. M. Coelho-Castelo

**Affiliations:** 1 Department of Biochemistry and Immunology, School of Medicine of Ribeirão Preto, University of São Paulo, São Paulo, Brazil; 2 Brazilian Tuberculosis Research Network of School of Medicine of Ribeirão Preto, University of São Paulo, São Paulo, Brazil; 3 Department of Cellular and Molecular Biology, School of Medicine of Ribeirão Preto, University of São Paulo, São Paulo, Brazil; 4 Division of Cell Biology and Immunology, School of Life Sciences, University of Dundee, Dundee, Scotland; Ordway Research Institute, United States of America

## Abstract

**Background:**

Experimental models using DNA vaccine has shown that this vaccine is efficient in generating humoral and cellular immune responses to a wide variety of DNA-derived antigens. Despite the progress in DNA vaccine development, the intracellular transport and fate of naked plasmid DNA in eukaryotic cells is poorly understood, and need to be clarified in order to facilitate the development of novel vectors and vaccine strategies.

**Methodology and Principal Findings:**

Using confocal microscopy, we have demonstrated for the first time that after plasmid DNA uptake an inhibition of the acidification of the lysosomal compartment occurs. This lack of acidification impaired antigen presentation to CD4 T cells, but did not alter the recruitment of MyD88. The recruitment of Rab 5 and Lamp I were also altered since we were not able to co-localize plasmid DNA with Rab 5 and Lamp I in early endosomes and late endosomes/lysosomes, respectively. Furthermore, we observed that the DNA capture process in macrophages was by clathrin-mediated endocytosis. In addition, we observed that plasmid DNA remains in vesicles until it is in a juxtanuclear location, suggesting that the plasmid does not escape into the cytoplasmic compartment.

**Conclusions and Significance:**

Taken together our data suggests a novel mechanism involved in the intracellular trafficking of plasmid DNA, and opens new possibilities for the use of lower doses of plasmid DNA to regulate the immune response.

## Introduction

The use of plasmid DNA in vaccinology and gene therapy has shown that plasmid DNA is efficient in generating humoral and cell-mediated immune responses to a wide variety of DNA-derived antigens in various animal models [1]. In the late 1980s, several groups demonstrated that intramuscular injection of naked DNA led to its transcription in myocytes resulting in the secretion of the encoded protein [2], [3]. Later studies found foreign gene expression after direct injection into other tissues such as heart, thyroid, skin and liver [4]–[7]. Despite the progress in DNA vaccine development, the intracellular transport and fate of naked plasmid DNA in eukaryotic cells is poorly understood. These details need to be clarified in order to facilitate the development of novel vectors and vaccine strategies.

Investigations of the intracellular route of plasmid DNA adsorbed on cationic polymer, cationic lipid or encapsulated in polymers have however been widely explored [8]–[10]. These studies suggested that formulation with positive charge such as lipoplex and polyplex were internalized by clathrin-dependent endocytosis [11]. However, the size, as well as the composition of the complex can influence the route of internalization. Large lipoplex (up to 500 nm) complexes preferentially enter the cell by receptor- and clathrin-independent endocytosis while the smaller complexes (<200 nm) could be internalized via coated pits through a non-specific, clathrin-dependent process [12]. The internalization of DNA entrapped in PLGA polymer occurs by phagocytosis (for microparticles, 1–10 µm) or through fluid phase pinocytosis and clathrin-coated pits (for nanoparticles, 70 nm) [13]–[15]. In these models, after its uptake, it is thought that plasmid DNA escapes to the cytoplasm, and that this process occurs through the disruption of the endo-lysosomal membrane. It is generally accepted that DNA/lipid cationic complexes fuse with the endosomal membrane allowing the escape of DNA into the cytosol [16]–[18]. The mechanism suggested for cationic polymers is a “proton sponge-mediated escape” [19], [20] by which the free plasmid DNA which enters the cytoplasm is transported to the nucleus, where it can then be transcribed.

However, in contrast to complexed plasmid DNA, the intracellular route of naked plasmid DNA, is poorly analyzed and restricted to the uptake process of the linear molecule or analysis of the localization of DNA in permeabilized cells [21]–[23]. Some experiments have demonstrated that the uptake of naked DNA by muscle cells occurs through caveolae, [24], [25] while in keratinocytes, the uptake of naked DNA occurs by macropinocytosis [26]. After the uptake, there is a generalized concept that DNA is localized in endosomes from which it can escape to the cytoplasm before fusion with lysosomes which could result in DNA degradation. Another possibility is that the DNA could escape to the cytoplasm even from lysosomes, and once in the cytosol, naked DNA could reach the nucleus to be transcribed [10], [27], [28]. In addition, the trafficking and cell activation by CpG motifs via TLR is well known, and for TLR9 MyD88 molecules has been shown to play an essential role in cell activation [29]–[31].

Our studies have focused on intramuscular delivery of naked plasmid DNA encoding *M. leprae* 65-kD heat shock protein as a vaccine against tuberculosis. We observed that this form of administration results in good immune induction, as well as protection against virulent *M. tuberculosis* challenge [32], [33]. In addition, in heavily infected mice, vaccination with Hsp65-encoding DNA can switch a relatively inefficient immune response that produces only bacterial stasis to an efficient response that kills bacteria [34], [35]. However, one of the principal problems in DNA vaccine strategy is the high amount of DNA needed to induce an efficient immune response due to the adverse environment for plasmid DNA in eukaryotic cells. Therefore, an understanding of intracellular plasmid DNA trafficking could help in reducing the DNA dose. In this study we analyzed the fate of naked DNA-Hsp65 in macrophages and dendritic cells, in order to address the mechanisms involved in intracellular DNA trafficking. We demonstrate unexpected results concerning plasmid-lysosome interactions and present original data that opens a novel way to use plasmid DNA to manipulate the immune response.

## Results

### The intracellular compartmentalization of plasmid DNA by J774 macrophage

**Figure 1:**
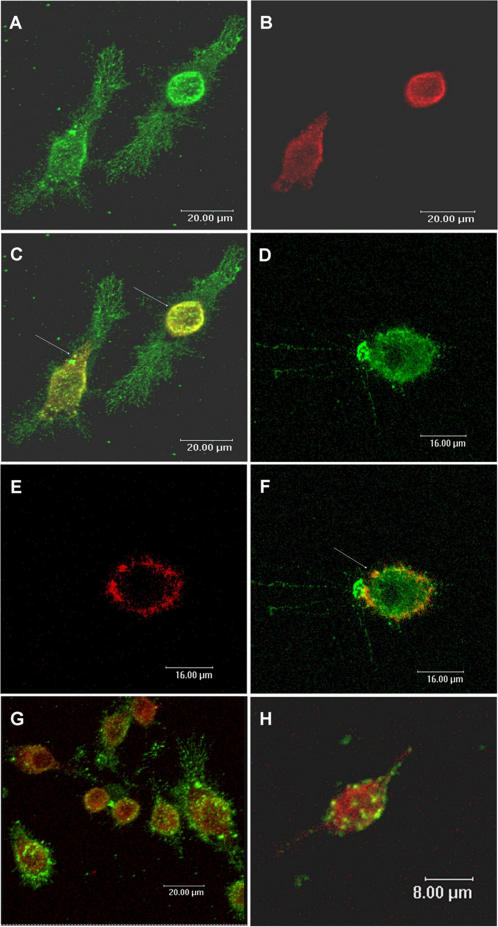
Visualization of plasmid DNA compartamentalization: the plasmid DNA co-localizes with transferrin in J774 cells. J774 cells were incubated with DNA-Alexa 488 (green) for 15 minutes (A and C) and 30 minutes (D and F). Transferrin conjugated to Alexa 594 (red) was used as marker of the early endosomes (B and E). While the plasmid DNA is colocalized with transferrin within 15 minutes (C, yellow, arrows), after 30 minutes, the plasmid DNA not was colocalized with these vesicles (F, arrow). (G–H): J774 cells were incubated with fluorescent DNA for 5 minutes (G) and 15 minutes (H); and cells immunostained for Rab5 (red), another marker of early endosomes. Plasmid DNA not was colocalized with Rab 5. Confocal images.

In order to determine the mechanism involved in plasmid DNA compartmentalization by eukaryotic cells we used J774 macrophages as a model. Transferrin, a marker of early endosomes, was used to investigate the presence of plasmid DNA in early endosomes (Figure 1). The results showed that in J774 cells, after 15 minutes, the plasmid DNA and the transferrin were colocalized in early endosomes in peripheral cytoplasmic compartments (Figure 1C). However, after 30 minutes, plasmid DNA no longer colocalized with early endosomes (Figure 1F). Even though the DNA was present in vesicles labeled with transferrin, suggesting that the uptake was receptor mediated in J774 cells, we did not observe co-localization with the early endosome marker Rab 5 after 5 or 15 minutes (Figures 1G and 1H).

### Plasmid DNA inhibits acidification of lysosomes

**Figure 2:**
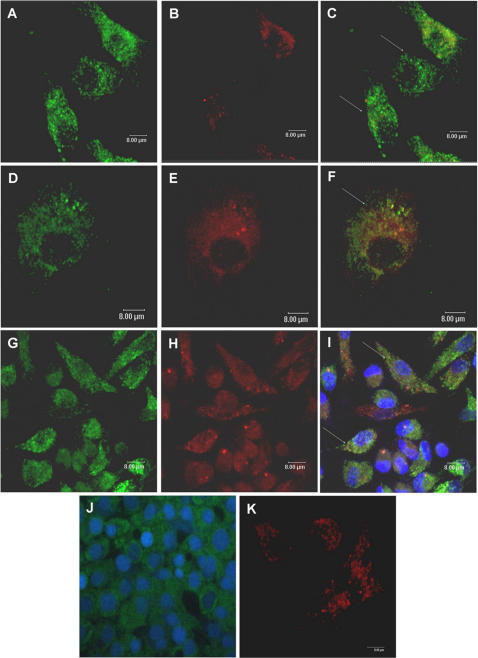
Intracellular trafficking of plasmid DNA. Confocal images of J774 cells (A–F) and peritoneal macrophages (G–I) incubated with DNA-Alexa 488 (green-A, D and G) for 24 hours (A–C; G–I) and 48 hours (D–F). LysoTracker Red was used as a marker for late endosomes and lysosomes (B, E and H). Plasmid DNA was not colocalized with LysoTracker Red which had a very weak signal (C, F and I). DAPI (Molecular Probes) was used to visualize nuclei (I and J). Note that the inhibition of acidification observed was not the result of fluorocrome interference, since we use plasmid DNA without labeling and there was no labeling with LysoTracker Red (J). (J): Unlabeled plasmid DNA plus LysoTracker Green. (K): Lysotracker Red without DNA.

**Figure 3:**
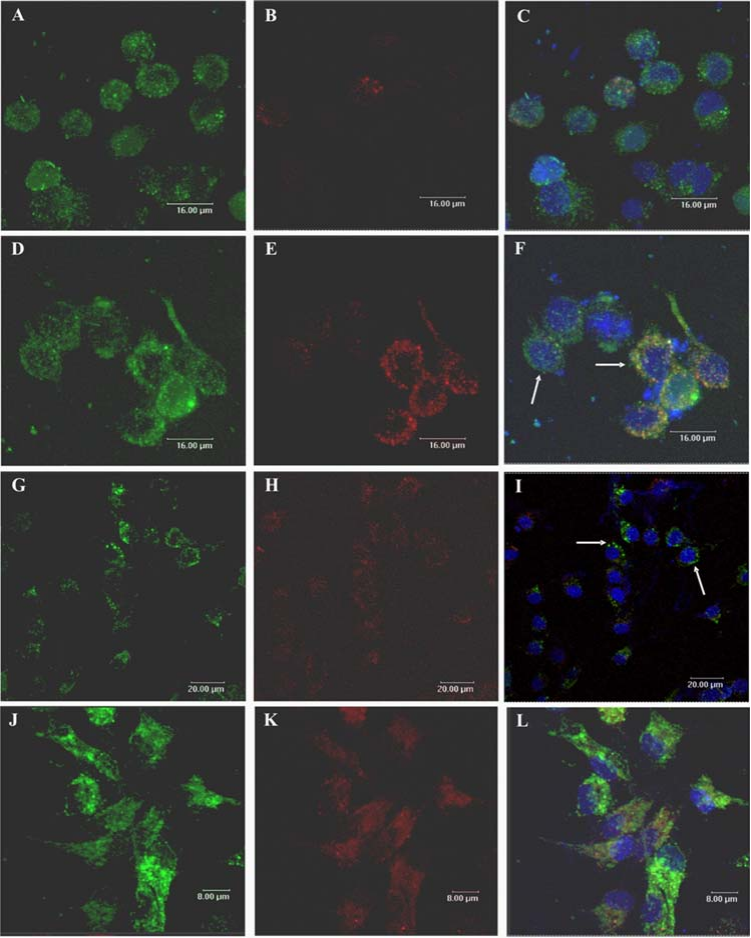
Intracellular trafficking of plasmid DNA. Confocal images of J774 cells (A–I) and peritoneal macrophages (J–L) incubated with DNA-Alexa 488 (green, A, D, G and J) for 72 h (A–C; J–L), 96 h (D–F) and 120 h (G–I). The LysoTracker Red was used as a marker of late endosomes and lysosomes (B, E, H and K). Plasmid DNA did not colocalize with LysoTracker Red's weak signal (C, F, I and L), and after 96 h and 120 h, fluorescent DNA was concentrated around the nuclei (F and I). DAPI (Molecular Probes) was used to visualize nuclei (C, F, I and L). In I cells are also seen in the Z axis.

**Figure 4:**
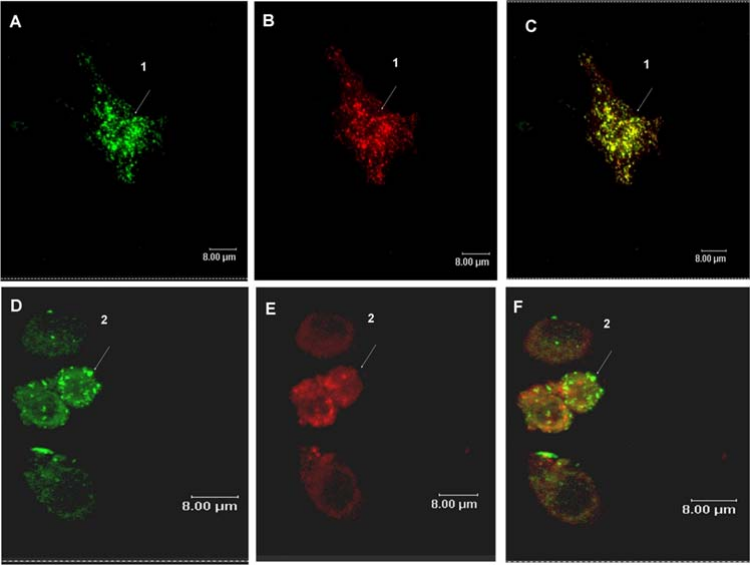
Intracellular trafficking of plasmid DNA. Confocal images of peritoneal macrophages incubated with DNA-Alexa 488 (green, A) for 24 hours. Texas Red dextran was used as lysosomal marker (B). Plasmid DNA was incubated with dextran and significant colocalization was observed (C, arrow 1). (D–F): J774 cells were incubated with DNA-Alexa 488 for 24 hours and the cells immunostained for LAMP I (red, lysosomal marker-E). Plasmid DNA did not colocalize with LAMP I (F, arrow 2).

Since plasmid DNA was no longer localized in early endosomes after 30 minutes, we investigated if the DNA was now present in late endosomes/lysosomes. To determine whether the plasmid was localized in these compartments, J774 cells were incubated with pcDNA_3_-Hsp65–Alexa 488 in the presence of LysoTracker Red, a marker for late endosomes and lysosomes. As shown in Figure 2 and Figure 3, fluorescent DNA was not co-localized with LysoTracker Red in the endo-lysosomal compartment at any of the times points analyzed (24 h–120 h). Similar results were observed in peritoneal macrophages, as showed in Figure 2 and Figure 3 (only 24 h and 72 h time points are shown). To confirm these results, pcDNA_3_-Hsp65-Alexa 488 was incubated with Texas Red dextran, a commonly used marker for lysosomes or endosomes depending on loading and chase times. Significant co-localization was detected after 24 h (Figure 4C), suggesting that the plasmid DNA was in lysosomes. The fact that plasmid DNA was not co-localized with LysoTracker Red, but was colocalized with Texas Red dextran, implies that when DNA is present in lysosomes it can interfere with the acidification of secondary endosomes/lysosomes (necessary for LysoTracker red accumulation). To eliminate the possibility that the observed inhibition of acidification was not the result of fluorocrome interference, we used unlabeled plasmid DNA and the results did not change (Figure 2J).

**Figure 5:**
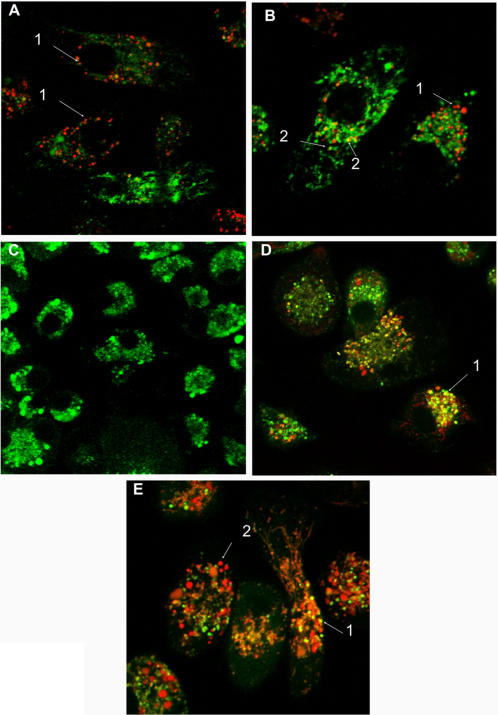
Visualization of the intracellular trafficking of plasmid DNA. Confocal images of spleen-derived dendritic cells (SDCs) incubated with DNA-Alexa 488 (green) for 1 hour (A–B). LysoTracker Red was used as marker for late endosomes and lysosomes. After the uptake the majority of the vesicles with plasmideal DNA were not colocalized with LysoTracker Red (A and B, -red, arrows 1). However, some colocalization was detected (B, yellow, arrows 2). (C–E): Visualization of the inhibition of the acidification of late endosomes/lysosomes by the plasmid DNA. Confocal images of spleen-derived dendritic cells (SDCs) incubated with DNA-Alexa 594 (red) and DQ Ovalbumin simultaneously (C: 1 min; D: 24 h and E: 72 h). DQ Ovalbumin is a self-quenched conjugate of ovalbumin that exhibits bright green fluorescence upon proteolytic degradation. It's was used as tool to determined inhibition of the acidification of late endosomes/lysosomes by the plasmid DNA. At 1 minute DQ Ovalbumin was degraded (green color) and the plasmid not was captured yet (not red color). After 24 h several colocalization points was observed (D-arrow 1) and after 72 h only some colocalization was detected (E-arrow 1), suggesting that DNA (red color, E-arrow 2) inhibited the acidification of the vesicles, prejudicing the OVA proteolytic degradation.

In the experiments using spleen-derived dendritic cells (SDCs), some colocalization of the DNA-Alexa 488 and LysoTracker Red was detected after 1 hour. However, only a few vesicles containing DNA were labeled with LysoTracker (Figures 5A and 5B). These results suggest that as seen with macrophages, plasmid DNA can inhibit lysosomal acidification in SDCs. To confirm these results, pcDNA_3_-Hsp65-Alexa 594 was incubated with DQ Ovalbumin, a self-quenched conjugate of ovalbumin that exhibits bright green fluorescence upon proteolytic degradation. As shown in Figure 5C, after 1 minute DQ Ovalbumin was degraded (green color) as the plasmid was not captured to any extent (not red color). After 24 h several colocalization points were observed (Figure 5D-arrow 1) and after 72 h less colocalization was detected (Figure 5E-arrow 1), suggesting that DNA uptake (Figure 5E-arrow 2) inhibited the acidification of the vesicles, preventing the OVA proteolytic degradation.

At all time points (1 h–120 h) DNA was localized in vesicles and after 96 h the vesicles containing plasmid DNA were localized around nuclei (Figure 3F and 3I). These results demonstrate that after endocytosis plasmid DNA remained in the vesicles until it arrived adjacent to the nucleus, suggesting that bulk of the DNA does not escape from the endo-lysososomes to the cytoplasm (Figures 3 and 4).

In spite of the fact that DNA was present in lysosomes we did not observe any colocalization, after 24 hours (Figures 4D and 4F), with Lamp I (lysosome associated membrane protein), a protein mainly localized in the limiting membranes of lysosomes and late endosomes.

### DNA treatment impairs KLH antigen presentation

**Figure 6:**
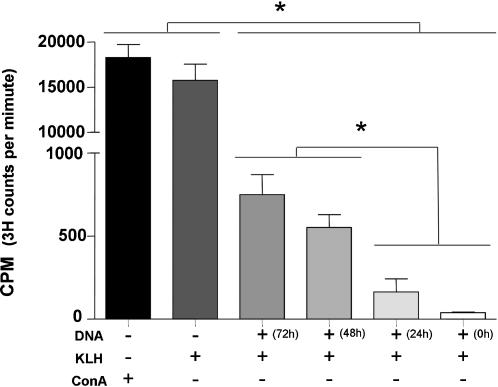
DNA treatment impairs KLH antigen presentation. Peritoneal macrophages were incubated with DNA (20 µg) 72 h, 48 h, 24 h or 0 h prior to the treatment with KLH (100 µg). After KLH treatment (24 h), the peritoneal macrophages were fixed and CD4 T cells specific for KLH were added to the culture. Proliferation was measured after 72 h. Concanavalin A (40 µg/ml) was used as positive control. * p<0.05 (OneWayANOVA).

To investigate the importance of the inhibition of the acidification of late endosomes/lysosomes by the plasmid DNA, the process of antigen presentation was analyzed. KLH antigen presentation was used as a model. The results showed that plasmid DNA completely inhibited the KLH antigen presentation when the DNA (20 µg) was added to the culture simultaneously with KLH, and antigen presentation was impaired when DNA was added at 24 h, 48 h or 72 h before exposure to KLH. The cells without plasmid DNA treatment showed antigen specific proliferation as a result of antigen presentation, while treatment with Con A (40 µg/ml), as a positive control, also resulted in proliferation (Figure 6).

### Plasmid DNA was internalized by TLR9 KO cells and Myd88 recruitment was not affected by pH of endososomes

**Figure 7:**
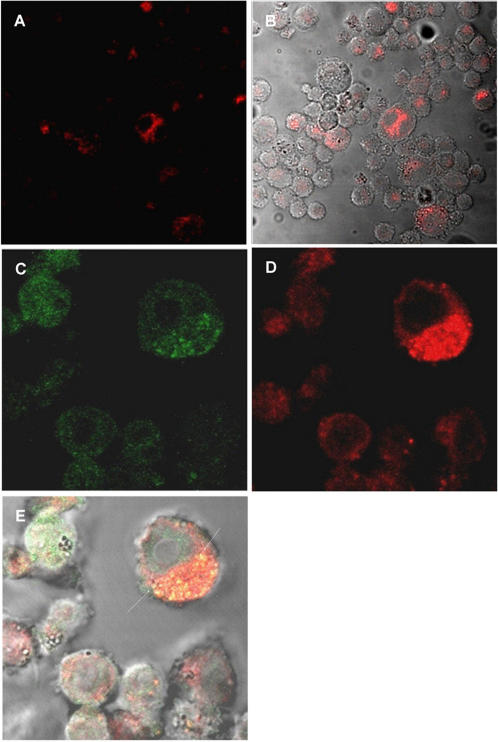
Uptake of DNA was independent of TLR9 receptor and recruitment of Myd88 was not affected. TLR9 KO cells were incubated with DNA-Alexa 594 (red-A and B) for 1 hour. The DNA was internalized by these cells. Raw cells (C–E) expressing GFP-tagged MyD88, were incubated with DNA-Alexa 594 (red-D) for 1 hour. Colocalization of GFP-Myd88 and fluorescent DNA was observed in vesicles (Arrows, yellow fluorescence–E).

As antigen presentation was altered in the presence of lower doses (20 µg) of plasmid DNA treatment (since in vaccination with DNA we used 300 µg of the plasmid DNA), we also investigated the involvement of myeloid differentiation factor 88 (MyD88) recruitment, classically triggered by CpG motifs present in plasmid DNA. The results demonstrated that DNA could be internalized by TLR9 KO cells and the recruitment of Myd88 to vesicles was not modified in Raw-Myd88GFP cells (Figure 7). These results suggest that the uptake of the plasmid DNA may be independent of TLR9 and alcalinization of endocytic vesicles does not interfere with MyD88 recruitment to the lysosomal membrane.

## Discussion

This work reports the basic mechanisms of intracellular DNA plasmid trafficking and opens new perspectives for the use of plasmid DNA in immune response control and vaccinology since it can modify the endosome/lysosome pH and interfere with antigen presentation. The normal endocytic processing of macromolecules in antigen presenting cells results in the internalization of specialized regions of the plasma membrane as well as small volumes of extracellular fluid. This process occurs by multiple mechanisms such as clathrin-mediated endocytosis, caveolae-mediated endocytosis, clathrin- and caveolae- independent endocytosis and macropinocytosis [36]. After internalization, macromolecules can traffic through the cell in endosome/lysosome compartments that can be identified by labeling of their intrinsic protein markers. For example, early endosomes can be identified by the presence of Rab5, and late endossome/lysosome by Lamp I [37], [38]. Plasmid DNA, in order to be transcribed and then activate the immune response, must surmount various cellular barriers such as the plasma membrane, endo-lysosomal compartments, transport through the cytoplasm and finally, the nuclear envelope. First, the naked DNA needs to be captured by an antigen presenting cell and internalized. This process, like others involved in the plasmid trafficking, is poorly understood. Wolff et al. [24] were the first to demonstrate that naked DNA is taken up by skeletal muscle via caveolae. Alternatively, macropinocytosis of naked DNA was showed in keratinocytes [26]. Our results suggest that in J774 cells the plasmid DNA appears to follow the same initial endocytic pathway as transferrin since the DNA was colocalized with transferrin. These results suggest that the endocytosis occurs via clathrin-coated pits [36], but that Rab 5 does not seem to be involved in the fusion and motility of the early endosomes containing plasmid DNA.

In addition, experiments with DNA and caveolin-coated pit markers showed no co-localization (data not shown). Clathrin mediated endocytosis was also recently described for uptake of CpG DNA by dendritic cells [39]. However, Ahmad-Nejad et al. [40] showed that endocytosis of CpG motifs was dependent on Rab5 for endosomal transport. Normally, Rab5 is required for homotypic fusion of early endosomes as well as endosomal vesicular trafficking [41]–[43]. The alteration in vesicle pH seen in the present experiments may explain the lack of involvement of Rab 5 seen with the plasmid DNA.

Our data demonstrated that after uptake of plasmid DNA, it moves out of early endosomes, but remains in vesicles until it arrives close to the nucleus, suggesting that the bulk of the plasmids do not escape into the cytoplasm.

This is the first time that this intracellular pathway has been reported for naked plasmid DNA. In delivery systems involving DNA complexed to synthetic carrier molecules, such as cationic lipids or polymers escape to the cytoplasm has been demonstrated. With cationic lipids as the carrier, the disruption of the endo-lysosomal membrane occurs through the interaction of positively charged cationic lipids with the negatively charged endosome/lysosome membrane, leading to membrane destabilization and allowing the penetration of DNA into the cytoplasm [16]–[18]. For cationic polymers, the suggested mechanism is a “proton sponge-mediated escape”. This theory proposes that cationic polymers have the ability to buffer endosomal pH thus causing an osmotic swelling of the endosomes, due to an increased influx of protons resulting in an influx of chloride ions and water [19], [20].

Unlike the cationic carriers, naked plasmid DNA has a negative charge similar to the endo-lysosomal membrane. The charge similarity would result in a lower association between the plasmid DNA and endo-lysosomal membrane, which then impedes the escape of the plasmid into the cytoplasm. Considering that cytoplasm is composed of a meshworks of cytoskeletal filaments, a variety of organelles and has a high protein concentration, the diffusion of large macromolecules such as DNA may be limited [44]. If the plasmid DNA stays in vesicles it may be able to move through the cytoplasm more easily. However, we have not yet elucidated the exact mechanism by which the plasmid DNA remains in the vesicles or the biological relevance of this. Importantly, we observed that the plasmid seems to inhibit the acidification of the late endosomes/lysosomes. The exact mechanism by which plasmid DNA influences the pH of the lysosome remains unknown, but this strategy presumably allows the plasmid DNA to avoid degradation by lysosomal enzymes including DNases [45], [46] and prevents a change in the plasmid conformation thereby reducing the efficiency of transcription. Moreover, the increase in lysosomal pH did not alter the uptake of DNA, since treatment with chloroquine also did not interfere with this process (Figure S1).

Using immature SDCs, we observed that the majority of the vesicles with plasmid DNA did not colocalize with LysoTracker, showing that, as previously described for macrophages, the alteration of pH occurs in various APC types. Moreover, the alteration of pH was also demonstrated when the incubation with DNA prevented the OVA proteolytic degradation (Figure 5). Also the vesicles containing plasmid DNA did not immunolable with the Lamp I (Figure 4), suggesting that the endosomal pH modification could interfere with the recruitment of this molecules to vesicles. Another possibility is that DNA could bind to Lamp I and interfere with its detection.

Furthermore, the lack of Rab5 and Lamp1 appears not to interfere with the migration of these vesicles to the peri-nuclear region. We also analyzed if the plasmid uptake was dependent on TLR9. Our results showed that the plasmid uptake was independent of the presence of TLR9 receptor. These results were expected, since studies showed that TLR9 is localized in the endoplasmatic reticulum of the dendritic cells and macrophages [39] and not localized in the plasma membrane. Since the cellular activation after CpG stimulation occurs via MyD88 recruitment, we analyzed if alcalinization of late endosomes/lysosomes could interfere with MyD88 recruitment. We could still detect recruitment of MyD88 to endosomes after plasmid treatment in spite the alteration in the pH of the vesicles suggesting that the signaling for recruitment of adaptor molecules was not changed. Although we cannot exclude some downstream interference in this signaling route, our results are in contrast with the work of Ahmad-Nejad et al. [40] which showed that vesicle acidification is a prerequisite to recruitment of MyD88. We believe that these different results could be due to the stimulus used. We were looking at cell activation using bacterial circular plasmid molecules, while Ahmad-Nejad et al. [40] used a linear stimulatory bacterial DNA (CpG motif).

Since our results demonstrated that plasmid DNA inhibits the acidification of late endosomes/lysosomes, we analyzed if this phenomenon could impair antigen presentation by class II MHC molecules. Our results demonstrate that the presentation of KLH antigen, by class II MHC, was impaired if the cells were incubated with low doses (20 µg) of DNA before or at the same time as KLH stimuli. Furthermore, the production of inflammatory cytokines stimulated with DNA was dose dependent, lower DNA doses producing less cytokines (data not showed). These results suggest that the DNA molecule could be used to control the immune response in inflammatory processes. It is known that proteolytic enzymes are responsible for degradation of protein antigens and this is dependent on the acidic pH of the endosome/lysosome compartment. Moreover, proteolytic enzymes also act on the invariant chain of the class II MHC molecules [47]. Since plasmid DNA inhibited the acidification of the late endosome/lysosomes, we hypothesize that this may also inhibit these proteolytic enzymes resulting in an absence of antigen degradation and impairment of KLH antigen presentation. Further studies are required to verify this hypothesis. Although these data were unexpected, they could change the way plasmid DNA is used in immune response activation.

Recently Accapezzato et al. [48] showed that the use of chloroquine, a lysosomotropic agent, improved the cross presentation in immature dendritic cells to CD8 T cells, by increasing the export of antigen from lysosomes to the cytosol. We did not analyze whether a cross presentation of antigen to CD8 T cells was present in our system, but we can not exclude this possibility, since the plasmid DNA interfered with the pH of the late endosomal/lysosomal compartment in different antigen presenting cells.

The current results imply a new intracellular route for plasmid DNA trafficking in vesicles to nuclei that may ensure that bacterial plasmids avoid intracellular degradation. However, the exact mechanism still remains to be elucidated. Taken together these results suggest a wide range of applications for naked DNA including studies on antigen presentation since these results suggest that lower plasmid DNA concentrations can be used to control the immune response (the use of lower doses of plasmid DNA to regulate the immune response was deposited with the patent number 0.700.698-5/I.N.P.I./SP).

## Materials and Methods

### Cell culture

The macrophage line Raw-Myd88GFP, TLR9 KO cells (both kindly provided by Dr. Hermann Wagner, Institute of Medical Microbiology, Immunology and Hygiene, Munich, Germany), the J774 macrophage line, peritoneal macrophages (collected from Balb/c mice by peritoneal lavage with PBS), and spleen-derived dendritic cells (SDCs) were used for studying the cellular uptake and trafficking of plasmid DNA. Dendritic cells (SDCs) were derived from the spleen of mice (Balb/c) and maintained, in low adherence plates, as described by West et al. [49], in RPMI medium supplemented with 10% fetal calf serum (Invitrogen, Gibco, Carlsbad, CA), 100 mM sodium pyruvate, 10 mM non essentials aminoacids, 10 mg/mL kanamicin, 200 mM glutamine, 10 mM β-mercaptoethanol, 10 ng/mL GM-CSF (R&D Systems, Minneapolis, USA) and 1 ng/mL TGF-β (R&D Systems, Minneapolis, USA) for 14 days (medium was replaced every 2 days) before use. The other cells (J774, Raw-Myd88GFP and peritoneal macrophages) were cultured in RPMI 1640 medium, supplemented with 10% FCS (Gibco), 100 UI/mL penicilin-streptomicin (Gibco) and 10 µg/mL gentamicin (Gibco). All cells were cultured at 37° in a humidified atmosphere of 5% CO_2_ in air.

### Plasmid

The plasmid pcDNA_3_-Hsp65 contained the cytomegalovirus (CMV) promoter and a cDNA encoding the gene for *M. leprae* heat shock protein 65 kDa which has previously been described (Lowrie et al., 1999). DH5 α E. coli transformed with pcDNA_3_-Hsp65 was cultured in LB liquid medium (Gibco, Grand Island, NY, USA) containing ampicilin (100 µg/mL, Cilinon™). The plasmid was purified using the Endo-Free QIAGEN plasmid purification kit (QIAGEN AG, Basel, Switzerland). Plasmid concentration was determined by spectrophotometry at λ = 260 and 280 nm using the Gene Quant II apparatus (Pharmacia Biotech, Buckinghamshire, UK). The purity of DNA preparations was confirmed by electrophoresis on a 1% agarose gel.

### Plasmid DNA labeling

Plasmid DNA was labeled with Alexa Fluor 488 or 594 by Universal Linkage System (ULS™) using the ULYSIS nucleic acid labeling kit (Invitrogen, Molecular Probes, Inc., Carlsbad, CA) with modifications as described by Coelho-Castelo et al [50]. Briefly, the plasmid DNA (8 µg) in labeling buffer (25 µL) was denatured at 95°C for 5 minutes and cooled on ice. The ULS labeling reagent stock solution (2.5 µL) was added to the tube and the reaction incubated at 80°C for 15 minutes. The labeled DNA was purified by ethanol precipitation, followed by resuspension in PBS. The conformation of labeled plasmid prepared in this way was not altered (data not shown). No free dye was associated with the DNA.

### Confocal laser microscopy

J774, Raw-Myd88GFP, peritoneal macrophages or SDCs were plated 24 h prior to the experiment on glass 8 well chamber slides (Lab-Tek Chamber Slide System, Nalge Nunc International, Rochester, NY) at 2×10^4^ cells/wells in RPMI medium, without phenol red, with 10% FCS. For the experiments involving Alexa 594 transferrin (14 µg/mL-Invitrogen, Molecular Probes, Inc., Carlsbad, CA), J774 cells were incubated with 4 µg of the Alexa Fluor 488-labeled pcDNA_3_-Hsp65 in medium containing Alexa 594 transferrin for 30 minutes or the marker was added first and after 15 minutes fluorescent DNA was added for an additional 15 minutes. For experiments involving LysoTracker Red (160 nM- Invitrogen, Molecular Probes, Inc., Carlsbad, CA), J774 cells, peritoneal macrophages or SDCs, were incubated with Alexa Fluor 488-labeled pcDNA_3_-Hsp65 (4 µg) for varying times (1 h–120 h) and LysoTracker Red was added for the last 30 minutes. For the DQ Ovalbumin experiments (Invitrogen, Molecular Probes, Inc., Carlsbad, CA) SDCs were incubated simultaneously with Alexa Fluor 594-labeled pcDNA_3_-Hsp65 (4 µg) and DQ Ovalbumin for varying times (1 min, 24 h and 72 h). For the Texas Red dextran experiments (10.000 MW, 10 µg/mL-Invitrogen, Molecular Probes, Inc., Carlsbad, CA), peritoneal macrophages were treated with Alexa Fluor 488-labeled pcDNA_3_-Hsp65 (4 µg) in growth medium containing Texas Red dextran for 24 hours. After the cells were incubated with fluorescent DNA plus Texas Red dextran, they were washed twice with PBS, fixed for 15 minutes with 2% paraformaldehyde in PBS, rinsed in PBS and coverslips mounted with Fluormount (EM Sciences, Hatfield, PA). For the Rab 5 (BD Transduction Laboratories, New Bedford, MA, USA) and Lamp I (Santa Cruz Biotechnology, Inc., Santa Cruz, California) experiments, J774 cells were treated with Alexa Fluor 488-labeled pcDNA_3_-Hsp65 (4 µg) for various times (5 min and 15 min for Rab 5; and 24 hours for Lamp I) and after treatment cells were washed twice with PBS, fixed for 15 minutes with 2% paraformaldehyde in PBS. rinsed in PBS and nonspecific antibody binding sites were blocked with 3% bovine serum albumin and donkey IgG in PBS for 1 hour. The cells were immunostained by incubating them with primary antibody (Rab 5 or Lamp I) in blocking buffer (PBS containing 1% bovine serum albumin) containing 0.01% saponin at room temperature for 45 minutes, rinsed and then incubated in blocking buffer with secondary antibody labeled with Alexa 594 at room temperature for 45 minutes. The J774, Raw-Myd88GFP and peritoneal macrophages were examined by confocal (Leica TCS SP2 AOBS, Leitz, Manheim, Germany), or in the case of the SDCs, by vital microscopy (LSM510META, Zeiss Germany). In order to visualize Myd88 recruitment, Raw-Myd88GFP cells were treated with Alexa Fluor 594-labeled pcDNA_3_-Hsp65 for 1 hour, after they were washed twice with PBS, cover slips mounted with Fluormount and examined by confocal microscopy (Leica TCS SP2 AOBS).

### Antigen presentation

BALB/c mice, 6 to 8 weeks old, were obtained from the Animal Facilities of the School of Medicine of Ribeirão Preto, University of São Paulo, and were maintained under standard laboratory conditions. 50 µg of Keyhole limpet haemocyanin (KLH, Sigma-Aldrich, St. Louis, MO, USA) in 50 µL Freund's complete adjuvant (Sigma-Aldrich) was administered by subcutaneous injection into each footpad. After 7 days another injection of 50 µg KLH in 50 µL Freund's incomplete adjuvant was administered into each footpad (total dose 200 µg of KLH). CD4+ T cells from draining lymph nodes were purified by magnetic beads (MicroBeads CD4-L3T4 Miltenyi Biotec GmbH, Germany) 7 days after completion of immunization. Peritoneal macrophages (collected from Balb/c mice by washing the peritoneal cavity with PBS) were used as antigen presenting cells. For the antigen presentation experiments, peritoneal macrophages (5×10^5^ cells) were treated with DNA (20 µg) for 0 h, 24 h, 48 h, or 72 h, prior to treatment with KLH (100 µg). After 24 hours exposure to KLH, peritoneal macrophages were fixed and CD4 T cells specific for KLH from immunized mice (5×10^5^ cells) was added to the culture. CD4 T cells were cultured for an additional 72 hours and proliferation during the last 16 hours was quantified by incubation with 0.5 µCi of 3H-thymidine (Amersham, Les Ulis, France). Cells were collected onto filters and radioactivity was measured in a scintillation counter (Beckman, Germany). Concanavalin A (Con A–40 µg/ml; Sigma, St. Louis, MO, USA) was used as positive control (T cell mitogen).

## Supporting Information

Figure S1 Treatment with chloroquine did not interfere with the uptake and trafficking of DNA. Confocal images of J774 cells incubated with DNA-Alexa 488 (green-A) or fluorescent DNA plus cloroquine (B) for 4 h. (C) J774 cells incubated with LysoTracker Red, a marker of late endosomes and lysosomes. (D): J774 cells incubated with cloroquine plus LysoTracker Red. As the cloroquine neutralizes the vesicles, the the Lysotracker Red does not accumulate in lysosomes. (E): J774 cells in (D) incubated with cloroquine plus LysoTracker Red visualized by differential interference contrast.
(5.63 MB TIF)
