# Supplementary figures and images for: Endocytosis of DNA-Hsp65 Alters the pH of the Late Endosome/Lysosome and Interferes with Antigen Presentation
Source: PLoS One. 2007 Sep 26;2(9):e923. doi: 10.1371/journal.pone.0000923 (PMC1976595; doi:10.1371/journal.pone.0000923)

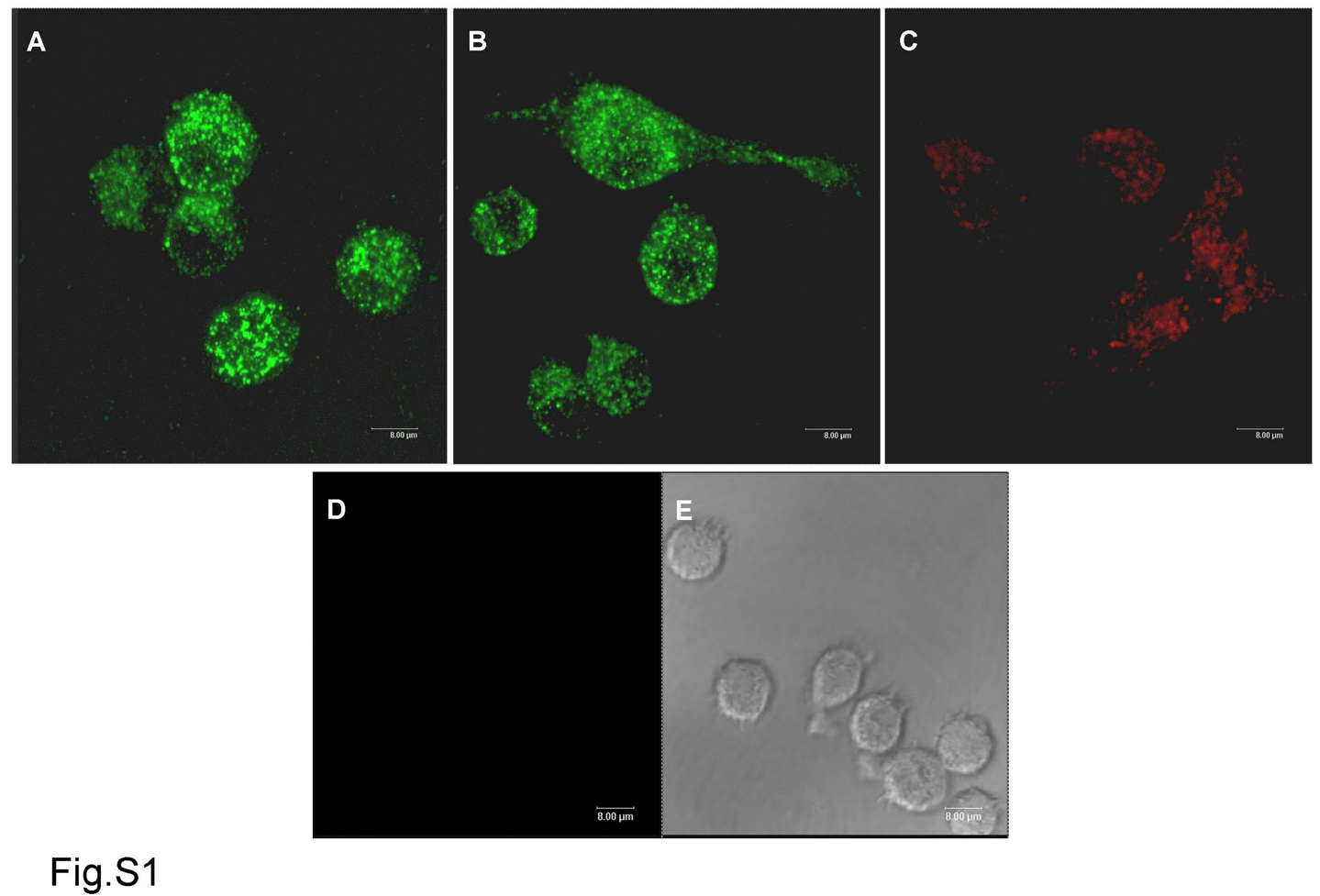

Supplement: Figure S1 — Treatment with chloroquine did not interfere with the uptake and trafficking of DNA. Confocal images of J774 cells incubated with DNA-Alexa 488 (green-A) or fluorescent DNA plus cloroquine (B) for 4 h. (C) J774 cells incubated with LysoTracker Red, a marker of late endosomes and lysosomes. (D): J774 cells incubated with cloroquine plus LysoTracker Red. As the cloroquine neutralizes the vesicles, the the Lysotracker Red does not accumulate in lysosomes. (E): J774 cells in (D) incubated with cloroquine plus LysoTracker Red visualized by differential interference contrast. (5.63 MB TIF) [file pone.0000923.s001.tif]
